# C1q/TNF-Related Protein 3 (CTRP-3) Deficiency of Adipocytes Affects White Adipose Tissue Mass but Not Systemic CTRP-3 Concentrations

**DOI:** 10.3390/ijms22041670

**Published:** 2021-02-07

**Authors:** Andreas Schmid, Martin Roderfeld, Jonas Gehl, Elke Roeb, Andrea Nist, Ho-Ryun Chung, Thorsten Stiewe, Thomas Karrasch, Andreas Schäffler

**Affiliations:** 1 Department of Internal Medicine III, University of Giessen, 35390 Giessen, Germany; Jonas.F.Gehl@med.uni-giessen.de (J.G.); thomas.karrasch@innere.med.uni-giessen.de (T.K.); andreas.schaeffler@innere.med.uni-giessen.de (A.S.); 2 Department of Gastroenterology, University of Giessen, 35390 Giessen, Germany; martin.roderfeld@innere.med.uni-giessen.de (M.R.); Elke.Roeb@innere.med.uni-giessen.de (E.R.); 3 Institute of Molecular Oncology and Genomics Core Facility, University of Marburg, 35037 Marburg, Germany; andrea.nist@imt.uni-marburg.de (A.N.); thorsten.stiewe@staff.uni-marburg.de (T.S.); 4 Institute of Medical Bioinformatics and Biostatistics, University of Marburg, 35037 Marburg, Germany; ho.chung@staff.uni-marburg.de

**Keywords:** CTRP-3 knockout, adipocyte, adipose tissue, adipokine, inflammation

## Abstract

CTRP-3 (C1q/TNF-related protein-3) is an adipokine with endocrine and immunological function. The impact of adipocyte CTRP-3 production on systemic CTRP-3 concentrations and on adipocyte biology is unknown. A murine model of adipocyte CTRP-3 knockout (KO) was established (via the *Cre/loxP* system). Serum adipokine levels were quantified by ELISA and adipose tissue (AT) gene expression by real-time PCR. Preadipocytes were isolated from AT and differentiated into adipocytes. Comparative transcriptome analysis was applied in adipocytes and liver tissue. Body weight and AT mass were reduced in CTRP-3 KO mice together with decreased serum leptin. In primary cells from visceral AT of KO mice, expression of adiponectin, progranulin, and resistin was induced, while peroxisome proliferator activated receptor γ (*PPARγ*) was decreased. M1/M2 macrophage polarization markers were shifted to a more anti-inflammatory phenotype. CTRP-3 expression in AT did not contribute to serum concentrations. AT and liver morphology remained unaffected by CTRP-3 KO. Myelin transcription factor 1-like (*Myt1l*) was identified as a highly upregulated gene. In conclusion, adipocyte CTRP-3 has a role in adipogenesis and AT weight gain whereas adipocyte differentiation is not impaired by CTRP-3 deficiency. Since no effects on circulating CTRP-3 levels were observed, the impact of adipocyte CTRP-3 KO is limited to adipose tissue. Modified AT gene expression indicates a rather anti-inflammatory phenotype.

## 1. Introduction

The adipose tissue represents the largest endocrine organ, affecting a wide spectrum of physiological processes at different anatomical locations [1,2]. Its endocrine impact is clearly based on the secretion of a plethora of “fat hormones” (adipokines). Several of these secretory hormones, such as leptin [3] and adiponectin [4,5], exhibit immunomodulatory properties and are therefore considered to connect immunological or inflammatory pathways with metabolic pathways (“metabolic inflammation”, “metaflammation”, “adipose inflammation”) [6,7,8].

A distinct set of adipokines increasingly gaining attention in this context is represented by the C1q/TNF-related protein (CTRP) family [7,9,10,11]. Among these adiponectin paralogs with a strongly conserved molecular structure, CTRP-3 can be considered as the best-characterized representative, with pleiotropic functions in cell proliferation [12], adipocyte biology [13,14,15], carbohydrate/lipid metabolism [16,17], and particularly in inflammation [18,19,20]. CTRP-3 attenuates inflammatory processes mediated by Toll-like receptors (TLRs). Thus, it might represent an antagonist of LPS-induced TLR4 signaling, as has been demonstrated in vitro and in vivo [15,21]. Of note, anti-inflammatory and anti-apoptotic functions of CTRP-3 in murine hippocampus and in microglial cells were reported recently [22]. Furthermore, Murayama et al. [20] reported elevated expression of CTRP-3 in murine models of rheumatoid arthritis and suggested a beneficial role of this adipokine in collagen-induced arthritis. By applying a murine and whole-body CTRP-3 knockout model, they observed an elevated expression of pro-inflammatory factors and a more severe phenotype in experimental arthritis, thus confirming the general anti-inflammatory character of CTRP-3. While focusing on immunological effects, Murayama et al. did not report any further significant physiological impact of CTRP-3 deficiency in their mouse model besides an elevated susceptibility to collagen-induced arthritis [20]. In contrast, neither systemic (whole-body) CTRP-3 deficiency nor CTRP-3 overexpression in transgenic mice had an impact on IL-1beta, IL-6, TNF-alpha, or MIP-2 induction in another study [19], which is contrary to previous findings based on recombinant CTRP-3 administration. Wolf et al. [23] described reduced liver size and elevated circulating interleukin-6 levels in whole-body CTRP-3 knockout mice fed a high-fat diet, without further impact on systemic glucose metabolism and insulin sensitivity. A very recent study [24] using another line of whole-body (systemic) CTRP-3 knockout mice reported decreased white adipose tissue mass in obese mice fed a high-fat diet. Further studies in murine CTRP-3 deficiency models reported rather contradictory results on CTRP‑3 regarding cardiac hypertrophy [25,26]. Importantly, an adipocyte-specific CTRP-3 knockout model has not yet been established.

The contradictory results of several studies mentioned above might be caused by different experimental approaches using recombinant CTRP-3, whole-body knockout, or transgenic overexpression, respectively. Since CTRP-3 is not exclusively expressed in adipocytes, the systemic and local consequences of a mainly adipocyte CTRP-3 knockout is of major interest with respect to adipocyte biology, adipocyte differentiation, adipose inflammation, metabolism, and polarization of monocytes/macrophages residing in adipose tissue. Since such an adipocyte knockout model has not yet been established, the present study therefore introduces a novel mouse model with an adipocyte CTRP-3 knockout and its basal phenotypical characterization under non-pathological conditions and normal chow. Since numerous studies in the literature focus on circulating CTRP-3 concentrations in mice and humans and since CTRP-3 is not exclusively expressed in adipocytes, it was one of our main aims to clarify the impact of adipocyte CTRP-3 gene expression on the respective CTRP-3 serum concentrations. Furthermore, the examination focuses on

- overall morphological and physiological development, especially adipose tissue and liver;
- differentiation and gene expression profile of adipocytes in different adipose tissue compartments;
- regulation of immunomodulatory and metabolically active adipokines, cytokines, and chemokines;
- comparative transcriptome analysis of mature adipocytes and total liver by RNA-Seq.

## 2. Results

### 2.1. Biometric Analysis of Adipocyte-Specific CTRP-3 KO Mice

#### General Phenotype and Body Weight

**Figure 1:**
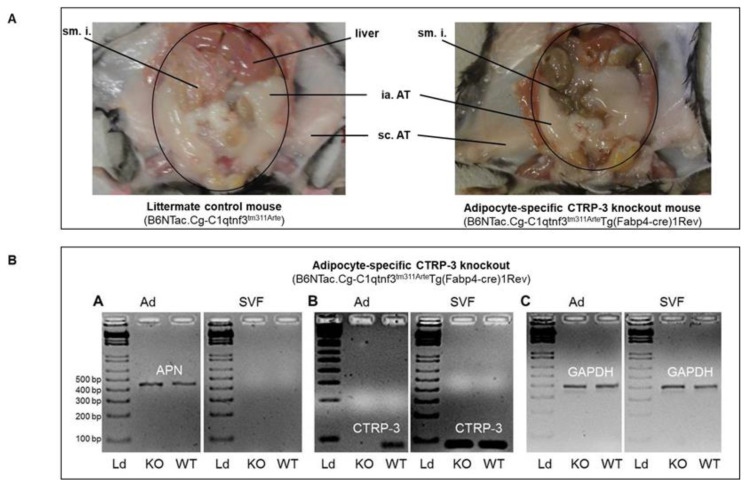
Generation of a novel adipocyte CTRP-3 knockout mouse model by using the transgenic *Cre/loxP* system with the *Cre* recombinase under the control of the *aP2* promoter. Panel (**A**): The macroscopic phenotype (abdominal situs) of littermate control mice and adipocyte CTRP-3 knockout mice was identical under basal conditions. sm.i., small intestine; ia. AT, intra-abdominal adipose tissue (yellow-white); sc. AT, subcutaneous adipose tissue (bright white). Panel (**B**): Gene expression in adipocytes and in stroma-vascular cells of littermate control mice (WT) and CTRP-3 KO mice. The adiponectin gene is expressed in adipocytes of control and KO mice but not in the stroma-vascular cell fraction (SVF) (B-A). The CTRP-3 gene is specifically knocked out in adipocytes of KO mice but not in wildtype mice or in the SVF (B-B). The expression of the housekeeping gene *GAPDH* was not altered (B-C). Mature adipocytes and cells of the stroma-vascular fraction were isolated from adipose tissue of CTRP-3 KO and control mice. After separation of cell fractions and isolation of mRNA, *C1qtnf3*, *APN* (adiponectin, as an adipocyte-specific marker), and *GAPDH* (as a housekeeping gene) were amplified by real-time PCR. Amplicons were visualized and analyzed by agarose gel electrophoresis. Ad, adipocyte; *APN*, adiponectin; *GAPDH*, glyceraldehyde-3-phosphate dehydrogenase; Ld, nucleic acid standard (DNA ladder); KO, adipocyte-specific CTRP-3 knockout mice (B6NTac.Cg-*C1qtnf3^tm311Arte^*Tg(Fabp4-cre)1Rev); WT, littermate control animals (B6NTac.Cg-*C1qtnf3^tm311Arte^*); SVF, stroma-vascular cell fraction.

Overall, KO mice did not obviously differ from control mice (Figure 1A) regarding skin and hair color, general behavior, locomotor activity, food intake, or fertility/reproduction in a large cohort of *n* = 127 mice. There was no evidence of disturbed or altered organogenesis or any clinical disease. At the age of 6 months, KO mice had a slightly but significantly decreased whole-body weight (*p* = 0.034) when compared to control animals (31.2 ± 5.5 g versus 33.4 ± 5.9 g) (Table 1).

### 2.2. Adipose Tissue Morphology

**Figure 2:**
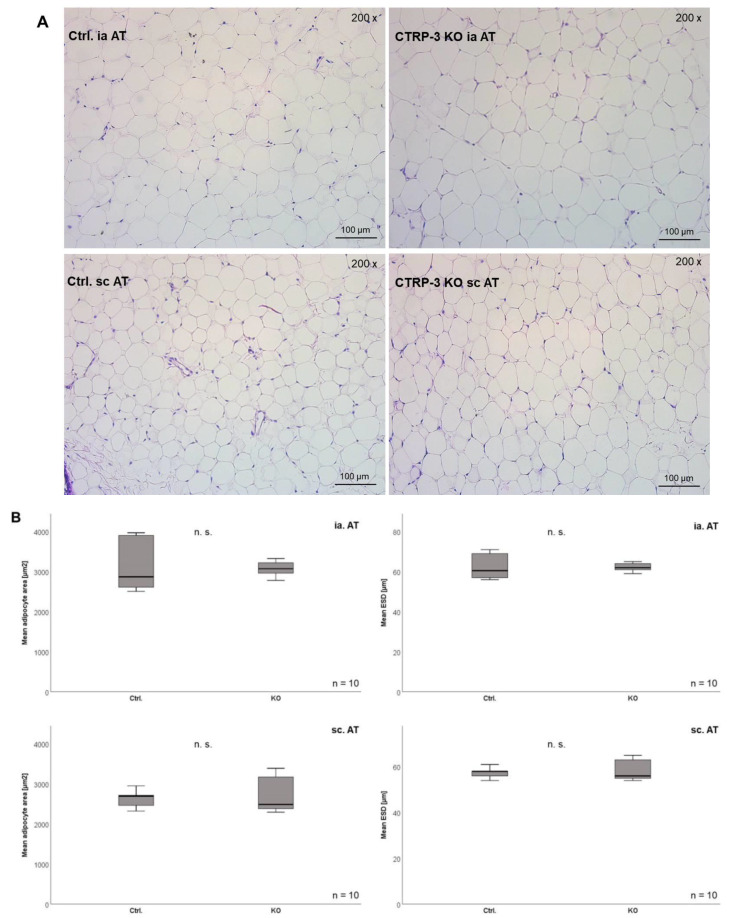
Histologic and quantitative examination of total subcutaneous and intra-abdominal adipose tissue cell morphology. Panel (**A**): Histologic slides after HE (hematoxylin–eosin) staining are shown (light microscopy; 200×). Panel (**B**): Quantitative analysis of mean adipocyte size (µm^2^) and mean adipocyte estimated standard diameter (ESD, µm). Ctrl., control animals; ia. AT, intra-abdominal adipose tissue; sc. AT, subcutaneous adipose tissue; KO, CTRP-3 knockout animals.

Importantly, the weights of intra-abdominal (visceral) adipose tissue (1.05 ± 0.68 g vs. 1.36 ± 0.68 g; *p* = 0.016), subcutaneous adipose tissue (0.92 ± 0.5 vs. 1.16 ± 0.56; *p* = 0.019), perirenal adipose tissue (0.48 ± 0.27 g vs. 0.64 ± 0.32 g; *p* = 0.006), and brown adipose tissue (0.28 ± 0.11 vs. 0.34 ± 0.13; *p* = 0.017) were significantly reduced in KO mice (Table 1). Figure 2A depicts histologic slides of intra-abdominal and subcutaneous adipose tissue, which were not altered concerning cell morphology and adipocyte size in KO mice when compared to control mice. As summarized in Figure 2B, quantitative analysis of mean adipocyte area (µm^2^) and mean estimated standard diameter (µm) showed no significant differences between genotypes, neither in intra-abdominal nor in subcutaneous adipose tissue.

### 2.3. Differentiation of Primary Preadipocytes into Mature Adipocytes

**Figure 3:**
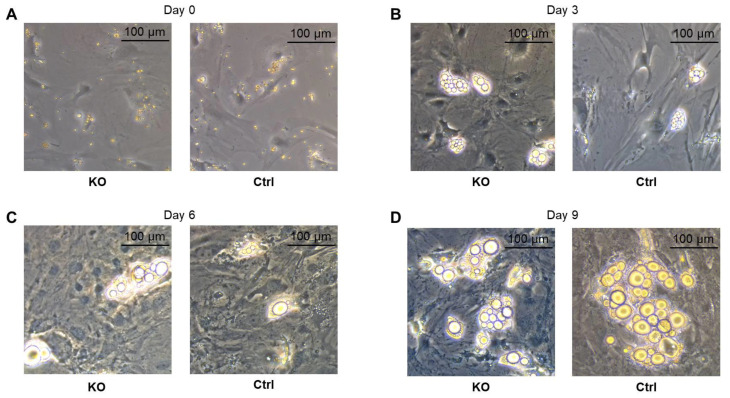
Differentiation of primary preadipocytes into mature adipocytes. Primary preadipocytes (**A**) were isolated from control littermates (Ctrl) and CTRP-3 KO animals (KO) and differentiated into mature adipocytes over 9 days by applying a hormonal differentiation protocol. Cellular phenotype is shown at days 3 (**B**), 6 (**C**), and 9 (**D**) of adipocyte differentiation (light microscopy, 200×). The process of differentiation and extensive lipid accumulation was apparently not altered by CTRP-3 KO.

In order to investigate whether the process of adipocyte differentiation and cellular lipid accumulation is disturbed by CTRP-3 KO per se, primary preadipocytes were isolated and differentiated in vitro into mature adipocytes over 9 days by applying a protocol of hormonally induced differentiation. As shown in Figure 3, adipocyte differentiation from fibroblast-like preadipocytes into mature adipocytes with extensive lipid accumulation was not altered.

### 2.4. Liver Morphology

**Figure 4:**
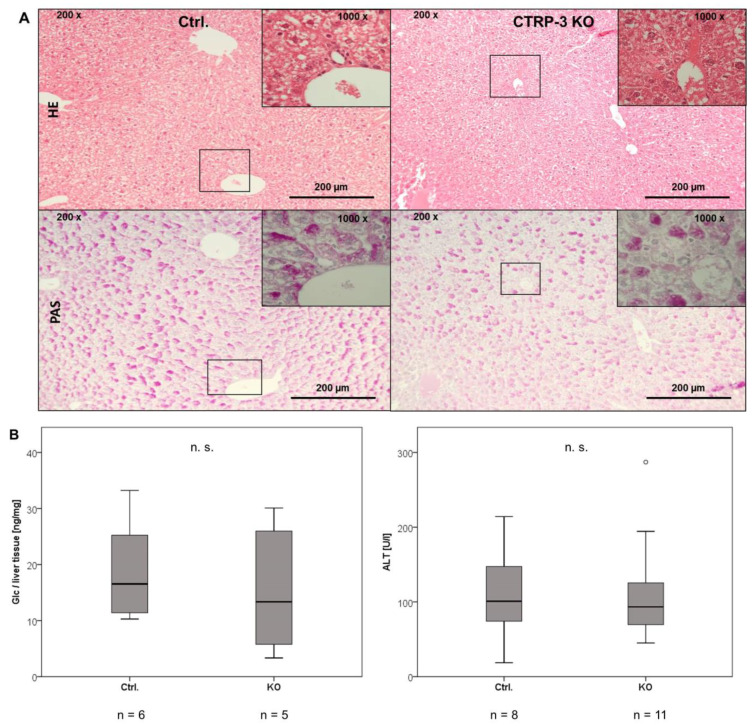
Histologic examination of liver cell morphology, glycogen content, and cell damage. Panel (**A**): Hepatic cell morphology (HE staining, light microscopy: 200× and 1000×) and glycogen storage (PAS staining, light microscopy: 200× and 1000×) is shown in control vs. KO animals. Panel (**B**): Quantitative analysis of liver glycogen content (quantification of supernatant glucose / liver tissue mass (ng/mg)) and serum ALT (U/L). ALT, alanine aminotransferase; Ctrl., control animals; Glc, glucose; HE, hematoxylin–eosin; KO, CTRP-3 knockout animals; PAS, periodic acid–Schiff reaction). °, outlier.

The average liver weight (Table 1) and liver integrity were unaffected by adipocyte CTRP-3 deficiency. Moreover, cell morphology and glycogen storage were not different in HE and PAS staining (Figure 4A) and there was no evidence of inflammation, fibrosis, or steatosis. Quantitative analysis of glycogen content and alanine aminotransferase (ALT) as a specific marker of hepatic injury (Figure 4B) revealed no differences between genotypes.

### 2.5. Serum Adipokine Concentrations in CTRP-3 KO Mice

Most importantly and unexpectedly, serum concentrations of CTRP-3 were equal in KO and control mice at the age of 6 months (KO: 5835 ± 857 pg/mL; Ctrl.: 5485 ± 1034 pg/mL) (Table 1), indicating that adipocyte-derived CTRP-3 does not represent the main cellular source for circulating CTRP-3 levels. Similarly, adiponectin, resistin, and progranulin serum levels were not significantly affected by adipocyte CTRP-3 deficiency (Table 1). Of note, systemic leptin concentrations were significantly decreased in KO mice compared to littermate controls (32.8 ± 22.7 ng/mL versus 47.2 ± 25.0 ng/mL; *p* = 0.048) (Table 1). Since leptin levels strongly decrease with weight loss and adipose tissue loss, this observation seems reasonable and expectable.

### 2.6. Adipokine and Cytokine Gene Expression Studies in Murine Adipose Tissue

Visceral adipose tissue specimens were obtained from euthanized mice bred under standard conditions and fed normal chow diet (age: 6 months) and mRNA was isolated for gene expression analysis by real-time PCR. As summarized in Table 1, gene expression of *adiponectin*, *leptin*, *resistin*, *progranulin*, and *PPARγ* were not influenced by CTRP-3 KO. The expression of the two lipases *ATGL* and *HSL* was also not different between control littermates and KO animals. Importantly, CTRP-3 KO had significant impact on the expression of M1/M2 macrophage polarization marker proteins (Table 1). Whereas the M1 polarization markers *TNFα*, *Il-6*, *RANTES*, and *CD80* remained unchanged, *MCP-1* expression was significantly decreased in KO mice (*p* = 0.013). Among M2 polarization markers, *CD206* was significantly increased (*p* = 0.005), whereas *IL-10* and *arginase-1* remained unchanged.

### 2.7. Adipokine Gene Expression in CTRP-3 Deficient Adipocytes

**Figure 5:**
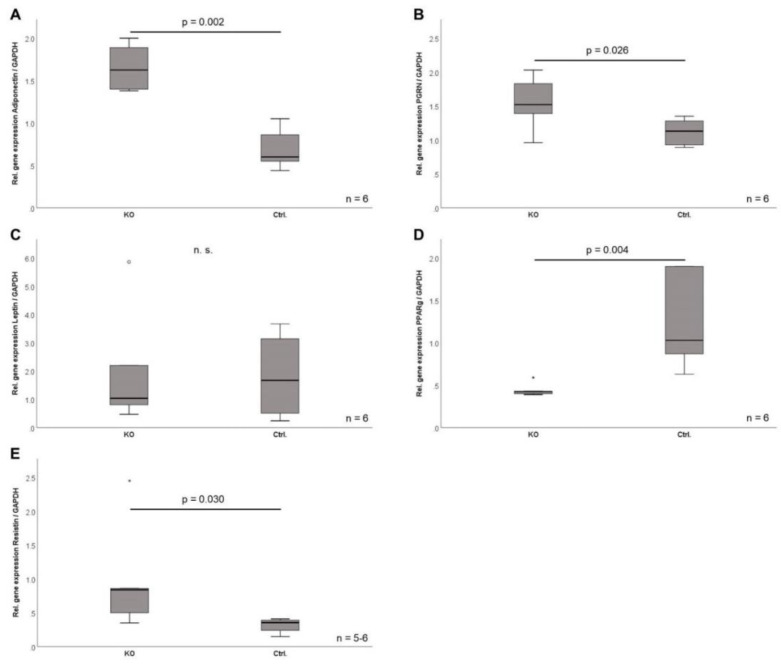
Gene expression analysis of adipokines in primary adipocytes. Preadipocytes (*n* = 6) were isolated from intra-abdominal adipose tissue of CTRP-3 KO mice and littermate control animals and were differentiated into mature adipocytes over 9 days of a hormonally induced differentiation program. Gene expression levels of adipokines and of the adipocyte-specific transcription factor *PPARγ* were determined in fully differentiated cells at day 9 by real-time PCR and were normalized to *GAPDH* mRNA levels. *GAPDH*, glyceraldehyde-3-phosphate dehydrogenase; *PGRN*, progranulin; *PPARγ*, peroxisome proliferator-activated receptor γ. °, outlier.

In order to investigate potential effects of CTRP-3 deficiency on a cellular level, primary preadipocytes were isolated from murine intra-abdominal and subcutaneous adipose tissue and were differentiated into mature adipocytes over 9 days ex vivo. The morphological process of preadipocyte differentiation was apparently not affected by CTRP-3 deficiency, as was determined by light microscopy (Figure 3). In primary cells derived from visceral adipose tissue of KO mice, gene expression analysis revealed significantly elevated *adiponectin* (*p* = 0.002), *progranulin* (*p* = 0.026), and *resistin* (*p* = 0.03) mRNA levels at day 9 during adipocyte differentiation (Figure 5). Moreover, *leptin* expression was unaffected, whereas *PPARγ* mRNA levels were significantly (*p* = 0.004) decreased (Figure 5) in KO animals. In primary cells derived from subcutaneous adipose tissue of KO mice, gene expression at day 9 of differentiation remained unchanged regarding *adiponectin*, *leptin*, *resistin*, *progranulin*, and *PPARγ*.

### 2.8. Transcriptome Sequencing of Isolated Mature Adipocytes in CTRP-3 KO vs. Control Mice

**Figure 6:**
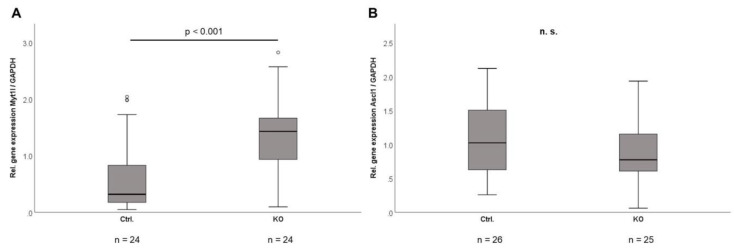
Gene expression analysis of *Myt1l* and *Ascl1* in adipose tissue. Intra-abdominal adipose tissue was obtained from CTRP-3 KO mice and littermate control animals. Gene expression levels were determined by real-time PCR and were normalized to *GAPDH* levels. *GAPDH*, glyceraldehyde-3-phosphate dehydrogenase; *Ascl1*, achaete-scute family BHLH transcription factor 1; *Myt1l*, myelin transcription factor 1-like. °, outlier.

Comparative transcriptome sequencing (CTPR-3 KO versus control mice) of isolated mature adipocytes from intra-abdominal adipose tissue revealed a very stable gene expression profile between genotypes under basal conditions in the absence of metabolic stress (Table 2A). Among the most significantly regulated genes, *Myt1l* (myelin transcription factor 1-like), *Mir6236* (mouse micro RNA 6236), and *Tef* (thyrotroph embryonic factor) were induced in KO animals. The expression of *Myt1l* together with its coregulatory transcription factor *Ascl1* (achaete-scute family BHLH transcription factor 1) was additionally analyzed by quantitative real-time PCR with specific primer pairs. As shown in Figure 6, adipose tissue from KO animals had significantly higher gene expression levels of *Myt1l*, whereas *Ascl1* remained unchanged. The putative context of these findings will be discussed later.

### 2.9. Transcriptome Sequencing of Total Liver Tissue from CTRP-3 KO vs. Control Mice

Comparative transcriptome sequencing (CTPR-3 KO versus control mice) of total liver tissue revealed a very stable gene expression profile between genotypes under basal conditions in the absence of metabolic stress (Table 2B). Among the downregulated genes, *complement factor D* (adipsin) playing a role in triacylglycerol synthesis and *perilipin-4* with its role in triglyceride droplet metabolism might be of special interest for the hepatic consequences of adipose tissue CTRP-3 KO. Among the upregulated genes, *EGFR* might be of future interest since it plays a role in the differentiation of adipocytes [27] and in the self-renewal of mesenchymal stem cells [28], which also orchestrate adipose tissue regeneration.

## 3. Discussion

Beside its pleiotropic endocrine and immunomodulatory effects, CTRP-3 has been suggested to play a crucial role in adipocyte differentiation in vitro [21]. Therefore, one substantial aim in phenotypical characterization of the novel murine model of adipocyte CTRP-3 deficiency was the identification of a potential impact on adipogenesis, adipose tissue weight, and whole-body weight. Biometric data were obtained from animals bred under standardized conditions and fed a standard chow for 6 months. Mice with adipocyte CTRP-3 knockout had a slightly (yet significantly) reduced whole-body weight when compared to littermate controls. Importantly, there was a significant reduction of intra-abdominal, subcutaneous, perirenal, and brown adipose tissue mass in KO animals. Along with these adipose tissue weight reductions, systemic leptin levels were decreased. Unlike leptin, there were no significant changes of the systemic levels of adiponectin, resistin, and progranulin, which has recently been shown to correlate positively with CTRP-3 serum levels in obesity [29].

In order to investigate the effects of adipocyte CTRP-3 deficiency on the cellular level, primary preadipocytes derived from murine intra-abdominal adipose tissue were differentiated into mature adipocytes ex vivo. The cellular process of differentiation and the extensive lipid accumulation typical for mature adipocytes were not altered by CTRP-3 deficiency. However, in primary cells derived from intra-abdominal adipose tissue of KO mice, gene expression analysis revealed elevated *adiponectin*, *progranulin*, and *resistin* mRNA levels at day 9 during adipocyte differentiation. Moreover, *leptin* expression was unaffected and *PPARγ* mRNA levels were decreased. In contrast, gene expression was not altered in primary adipocytes from subcutaneous adipose tissue. The observed changes of gene expression levels in intra-abdominal adipose tissue might be caused by reduced adipose tissue mass but seem to be too moderate to spill over into the systemic circulation. The downregulation of *PPARγ* as a main regulator of adipocyte differentiation might represent a mechanism contributing to reduction of total adipose tissue mass, although it apparently did not affect adipocyte differentiation and phenotype.

Comparative transcriptome analysis of mature adipocytes revealed a relatively stable gene expression profile among genotypes, with the identification of the zinc finger transcription factor *Myt1l* as one of the strongest upregulated genes in intra-abdominal adipocytes. This finding could be verified for total adipose tissue in distinct experimental settings by using quantitative real-time PCR. The upregulation of *Myt1l* in mature adipocytes might be a compensatory mechanism during adipose tissue loss and in response to declining leptin levels. Since intra-abdominal adipose tissue represents a densely innervated tissue and since *Myt1l* activates the transdifferentiation of adipocyte precursor cells (APCs) into functional neurons [30,31], one could speculate that it is activated in order to compensate for adipose tissue loss. However, future mechanistic studies are needed to address this speculation properly. Alternatively, CTRP-3 might represent an autocrine and paracrine inhibitor of *Myt1l* under physiological conditions, which could explain its induction upon adipocyte CTRP-3 KO. However, data on putative implications of CTRP-3 for *Myt1l* expression are completely lacking. Direct conversion of adipocyte progenitors into functional neurons requires the two transcription factors *Myt1l* and *Ascl1* [31]. Since *Myt1l* was strongly upregulated, one could speculate that adipocyte KO of CTRP-3 might create a microenvironment that facilitates neuronal (trans-) differentiation of precursors and/or compensatory processes of adipose tissue innervation or remodeling. However, this hypothesis is highly speculative—yet intriguing—and has to be investigated further.

Most interestingly, circulating CTRP-3 concentrations were found to be completely unaffected by adipocyte CTRP-3 deficiency. This observation clearly indicates that other cellular sources (such as the pancreas, small intestine, colon, brain, kidney, thymus, and ovaries) exist [11] that determine the systemic CTRP-3 concentrations. It therefore appears reasonable to assume that the primary effects of adipocyte CTRP-3 deficiency might be limited to adipose tissue (autocrine or paracrine regulatory pathways). The expression and secretion of CTRP-3 by adipocytes plays no role with respect to the respective serum concentrations, at least in mice. This is a novel and important finding and we therefore suggest that CTRP-3 should no longer be regarded as a classical adipokine.

The primary aim of the present study was to provide a basal characterization of adipocyte CTRP-3 knockout mice in order to facilitate the interpretation of available studies applying whole-body knockout, transgenic overexpression, or recombinant CTRP-3 administration, as summarized within the introduction. In contrast to whole-body knockout models, the application of the *aP2/FABP4* promoter-based *Cre/loxP* system has the advantage that CTRP-3 is knocked out specifically in adipocytes but not in the stroma-vascular cells of total adipose tissue. Concerning white adipose tissue mass, the present study on CTRP-3 KO fits well with a recent study investigating the consequences of a whole-body (systemic) KO in obese mice fed a high-fat diet [24]. Future studies are underway in order to describe the metabolic phenotype of our novel adipocyte CTRP-3 knockout model under metabolic stress such as high-fat diet and/or carbohydrate-enriched diet. Both models should then be compared in order to define the metabolic differences and consequences between adipocyte-specific and whole-body CTRP-3 deficiency. The present study further shows that adipose tissue and liver morphology were equal in KO and control mice—at least at the age of 6 months under basal conditions and in the absence of metabolic stress.

Considering the immunomodulatory functions of CTRP-3 in adipose tissue, the potential impact of adipocyte CTRP-3 deficiency on the inflammatory status of adipose tissue was of particular interest in the present study. Gene expression analysis investigating inflammatory chemokine and cytokine mRNA levels revealed significantly reduced adipose tissue *MCP-1* expression in KO mice. This is an important finding since MCP-1 represents a chemoattractant protein with a significant role in monocyte recruitment to adipose tissue and in pro-inflammatory polarization of resident macrophages. Furthermore, gene expression levels of macrophage polarization markers revealed an upregulation of *CD206* mRNA, a marker of anti-inflammatory (“M2”) polarization. On the other hand, gene expression of the pro-inflammatory (“M1”) macrophage marker *CD80* was apparently unaffected by adipocyte CTRP-3 KO. Taken together, adipocyte-specific CTRP-3 knockout resulted in a rather anti-inflammatory state of intra-abdominal adipose tissue when compared to control animals. The data suggest a rather beneficial immunomodulatory impact of CTRP‑3 KO and should encourage future experimental settings in order to investigate the implications of adipocyte CTRP-3 deficiency for the inflammatory state of resident immune cells, such as monocytes/macrophages and lymphocyte subsets, under metabolic stress.

## 4. Materials and Methods

### 4.1. Quantification of Adipokine Concentrations in Murine Serum

Concentrations of adipokines in murine serum were measured in duplicates by ELISA (CTRP-3: LSBio, Seattle, WA, USA; adiponectin, leptin, progranulin, resistin: DuoSet ELISA development systems, R&D Systems, Wiesbaden, Germany) and are expressed as mean values ± standard deviation. Measurement was generally repeated for samples exceeding an intra-duplicate variation of 20%. The lower detection limits were 15.6 pg/mL for resistin, 31.2 pg/mL for adiponectin and CTRP-3, and 125 pg/mL for leptin and progranulin.

### 4.2. Adipose Tissue and Adipocyte mRNA Extraction

Total RNA was isolated from adipose tissue using TRIzol^®^-Reagent (Life Technologies GmbH, Darmstadt, Germany) in combination with gentleMACS dissociator and M-tubes (Miltenyi Biotec GmbH, Bergisch Gladbach, Germany) for dissociation. RNA was isolated from prepared tissue using RNeasy^®^ Mini Kit (Qiagen, Hilden, Germany) including DNase digestion (RNase-Free DNase Set, Qiagen, Hilden, Germany) and gene expression was quantified by reverse transcription of mRNA (QuantiTect Reverse Transcription Kit from Qiagen, Hilden, Germany) and subsequent real-time PCR (iTaq Universal SYBR Green Supermix, CFX Connect RT-PCR system; Bio-Rad, Munich, Germany) of the corresponding cDNA as mentioned below in detail.

### 4.3. Real-Time PCR Analysis of Adipose Tissue mRNA Expression

Gene expression levels of target genes in adipose tissue compartments were determined by reverse transcription of isolated RNA and subsequent real-time PCR. Differences and changes in gene expression levels were calculated applying the delta-delta Ct method. The applied murine primer sequences are summarized in Table 3.

Expression levels of target genes were normalized to gene expression of murine *GAPDH* (isoform 1; NCBI Reference Sequence: NM_001289726.1) using a well-established primer pair that has been successfully applied for valid gene expression normalization in adipocytes in previous studies [13].

All oligonucleotides were purchased from Metabion, Martinsried, Germany.

### 4.4. Animals

#### 4.4.1. Adipocyte-Specific CTRP-3 Knockout

Wildtype (C57BL/6) and transgenic mice with adipocyte-specific CTRP-3 knockout (full nomenclature: B6NTac.Cg-*C1qtnf3^tm3113Arte^*Tg(*Fabp4-cre*)1Rev; abbreviation: CTRP-3 KO) together with littermate control mice (B6NTac.Cg-*C1qtnf3^tm3113Arte^*) were bred under standard conditions, fed a standard chow, and were euthanized for organ and tissue resection (Figure 1A). Intra-abdominal (visceral), subcutaneous (pooled from total dorsal and lateral body regions), perirenal (bilateral), and brown (interscapular) adipose tissue was resected. Adipose tissue specimens were either used for primary cell isolation or otherwise shock-frosted in liquid nitrogen. In the knockout model, adipocyte-specific deletion of exon 4 and subsequent frame-shift mutations within the *C1qtnf3* gene were introduced applying the *Cre/loxP* system, resulting in a dysfunctional gene product. Cell-type specificity of the knockout was achieved by transcriptional control of the *Cre* recombinase encoding transgene by adipocyte-specific *aP2*-promoter (from *Fabp4* gene). B6NTac.Cg-*C1qtnf3^tm3113Arte^* mice carrying *C1qtnf3* gene alleles with *loxP*-flanked exon 4 were created in collaboration with Taconic Artemis (Cologne, Germany). B6.Cg-Tg(*Fabp4-cre*)1Rev/J mice (purchased from Jackson Laboratories) were back-crossed to the C57BL/6NTac genetic background applying the Speed Congenics method for at least 6 generations, resulting in the strain B6.Cg-Tg(Fabp4-cre)1Rev/N. This offspring was crossed with C57BL/6NTac-*C1qtnf3^tm3113Arte^* mice in order to generate B6NTac.Cg-*C1qtnf3^tm3113Arte^*Tg(*Fabp4-cre*)1Rev mice with adipocyte-specific knockout of CTRP-3 (CTRP-3 KO).

For animal studies, an announcement was made at the local Ethical Committee (Regierungspräsidium Giessen) conformable to §4 Abs. 3 Tierschutzgesetz on 29 September 2014. Internal project identification code 544_M was assigned to the project at the University of Giessen.

Cell-type specificity of the CTRP-3 knockout was verified by mRNA extraction from separated adipocyte and stroma-vascular cell fractions derived from murine adipose tissue, followed by reverse transcription and real-time PCR using primers for CTRP-3 cDNA amplification. As visualized by agarose gel electrophoresis, CTRP-3 expression was specifically knocked out in mature adipocytes but not in stroma-vascular cells (Figure 1B).

#### 4.4.2. Analysis of Hepatic and Adipose Tissue Integrity

In murine livers and adipose tissues, histological analysis (hematoxylin–eosin (HE) staining) was performed in order to determine cell morphology and to exclude pathological conditions such as fibrosis, inflammation, and steatosis.

### 4.5. Glycogen Analysis

Quantification of hepatic glycogen was performed as described before [32]. Briefly, shock-frosted liver was grounded under liquid nitrogen. Then, 20 mg of the liver powder was hydrolyzed in 2M HCl in an Eppendorf tube at 95 °C for 60 min and subsequently neutralized with 0.5 M NaOH. After centrifugation at 14,000× *g* for 10 min, glucose was quantified in the supernatant using the Glucose (HK) Assay Kit as recommended by the manufacturer (Sigma-Aldrich, Deisenhofen, Germany, #GAHK20, #G3293). Periodic acid–Schiff (PAS) reaction was applied for visualization of glycogen storage in hepatic tissue.

### 4.6. Isolation and Cell Culture of Primary Murine Adipocytes

For primary cell culture, fresh visceral and subcutaneous adipose tissue obtained from CTRP‑3 KO mice was cut into small pieces and treated with 0.225 U/mL collagenase NB6 (Serva) at 37 °C for a maximum of 60 min. Digestion process was stopped by adding twice the amount of buffer (PBS containing 0.5% BSA and 2 mM EDTA). Cell suspension was filtered by 120 µm nylon mesh to eliminate undissolved tissue. Preadipocytes were separated from adipocytes by 10 min centrifugation at 300 g and 4 °C. Magnetic labeling plus depletion of non-adipocyte progenitor cells was done according to the manufacturer’s instructions (Adipose Tissue Progenitor Isolation Kit mouse, MACS Miltenyi Biotec, Bergisch Gladbach, Germany), as well as magnetic labeling and positive selection of adipocyte progenitor cells. Isolated preadipocytes were seeded at a density of 2.03 × 10^4^ cells/cm^2^ in DMEM (Dulbecco’s Modified Eagle Medium, Biochrom AG, Berlin, Germany) that was supplemented with 10% newborn calf serum (NCS; from Sigma-Aldrich, Deisenhofen, Germany) and cultured at 37 °C and 5% CO_2_. Adipocyte differentiation was initiated after cells reached 85% confluence. Cells were differentiated into adipocytes at confluence by DMEM/F12/glutamate medium (Lonza, Basel, Switzerland) supplemented with 20 µM 3-isobutyl-methyl-xanthine (Serva, Heidelberg, Germany), 1 µM corticosterone, 100 nM insulin, 200 µM ascorbate, 2 µg/mL transferrin, 5% fetal calf serum (FCS, Sigma-Aldrich, Deisenhofen, Germany), 1 µM biotin, 17 µM pantothenic acid, 1% penicillin/streptomycin (all from Sigma Aldrich, Deisenhofen Germany), and 300 µg/mL Pedersen fetuin (MP Biomedicals, Illkirch, France) [33,34] for 9 days using a slightly modified protocol as reported in the literature [35,36,37,38,39]. Phenotype was controlled by light-microscopy (appearance of extensive accumulation of lipid droplets). Cell phenotype during adipocyte differentiation was monitored by light microscopy.

### 4.7. Comparative Transcriptome Analysis of Single-Cell Adipocytes and Liver Tissue

mRNA was isolated from mature adipocytes derived from intra-abdominal adipose tissue and from total liver tissue of control and CTRP-3 KO mice. RNA integrity was assessed on an Experion StdSens RNA Chip (Bio-Rad). RNA-seq libraries were prepared using the TruSeq Stranded mRNA Library Prep kit (Illumina Inc., San Diego, CA, USA). Libraries were quantified on a Bioanalyzer (Agilent Technologies) and sequenced on an Illumina HiSeq 1500 platform, rapid-run mode, single-read 50 bp (HiSeq SR Rapid Cluster Kit v2, HiSeq Rapid SBS Kit v2, 50 cycles) according to the manufacturer’s instructions. Sequenced reads were aligned against Ensembl *Mus musculus* reference genome (ENSMUSG) (revision 83) applying Aligner STAR (version 2.4.1a). Aligned reads were counted by an in-house procedure, with reads from protein-coding genes selectively being counted in case of correct strand information and location in exon regions of the respective gene. Differential gene expression analysis was performed applying DESeq2 (version 1.12.3) and results were filtered for an FPKM (fragments per kilobase of exon model per million reads mapped) value > 0.3 and a minimum tag count > 50 in at least one sample. Genes were considered as differentially regulated in case of an absolute value of log_2_ FC (log_2_ fold change) > 0.5 and a corrected *p* value (false discovery rate fdr; Benjamini-Hochberg correction) < 0.05. For liver tissue samples, only results with log_2_ FC > 0.8 are shown for reasons of better clarity.

### 4.8. Statistical Analysis

For statistical analysis of data, a professional software program was used (SPSS 27.0, IBM Corp., Armonk, NY, USA). Nonparametric numerical parameters were analyzed by the Mann-Whitney U-test (for 2 unrelated samples), the Kruskal–Wallis test (>2 unrelated samples), the Wilcoxon test (for 2 related samples), or the Friedman test (>2 related samples). A *p*-value below 0.05 (two-tailed) was considered as statistically significant. Box plots are shown indicating median, upper/lower quartiles, interquartile range, minimum/maximum values, and outliers (indicated by “°”). Mean values ± SD are given for quantitative biometric parameters and adipokine serum levels. For gene expression analysis, mRNA levels were normalized to *GAPDH* expression and relative changes between control and KO mice were calculated applying the delta-delta Ct method and Mann–Whitney U-test.

## 5. Conclusions

The application of the *aP2/FABP4* promoter-based *Cre/loxP* system allowed us for the first time to investigate the molecular consequences of a specific CTRP-3 knockout in adipocytes without affecting CTRP-3 expression in the stroma-vascular cell fraction of total adipose tissue. The presented data argue for a role of adipocyte CTRP-3 in adipogenesis and age-related adipose tissue weight gain, whereas liver morphology remained unchanged. On the cellular level, adipocyte differentiation was found to be not impaired by CTRP-3 deficiency, suggesting compensatory mechanisms were rescuing the normal adipocyte phenotype. Since no significant effects on circulating CTRP-3 levels were observed, the physiological impact of adipocyte CTRP-3 knockout appears to be limited to adipose tissue. Thus, CTRP-3 should no longer be regarded as a classical secretory adipokine. Comparative transcriptome analysis of mature adipocytes revealed a relatively stable gene expression profile among genotypes, with the identification of *Myt1l* as one of the strongest upregulated genes. Modified gene expression in adipose tissue indicates a rather anti-inflammatory phenotype being associated with adipocyte CTRP-3 deficiency.

## Figures and Tables

**Table 1:** Tissue weights, adipokine serum concentrations, and visceral adipose tissue gene expression profiles in control vs. CTRP-3 knockout mice.

Tissue Weight	Control Mice *n*		CTRP-3 KO Mice *n*		*p*
Total body weight (g)	33.4 ± 5.9	65	31.2 ± 5.5	62	0.034 *
Intra-abdominal adipose tissue (mg)	1361 ± 678	61	1051 ± 677	59	0.016 *
Subcutaneous adipose tissue (mg)	1163 ± 561	60	915 ± 500	55	0.019 *
Perirenal adipose tissue (mg)	637 ± 315	60	475 ± 266	53	0.006 *
Brown adipose tissue (mg)	342 ± 133	60	284 ± 109	56	0.017
Liver weight (mg)	1600 ± 300	62	1568 ± 314	59	n.s.
Adipokine serum concentrations					
CTRP-3 (pg/mL)	5485 ± 1034	12	5835 ± 857	14	n.s.
Adiponectin (µg/mL)	7.35 ± 2.30	30	6.59 ± 2.18	28	n.s.
Leptin (ng/mL)	47.2 ± 25.0	28	32.8 ± 22.7	26	0.048 *
Resistin (ng/mL)	91.0 ± 33.4	30	100.0 ± 52.9	29	n.s.
Progranulin (ng/mL)	979 ± 382	65	983 ± 347	61	n.s.
Quantitative gene expression in visceral adipose tissue					
Adipokines/adipocyte differentiation					
*Adiponectin*		29	unchanged	28	n.s.
*Leptin*		29	unchanged	28	n.s.
*Resistin*		29	unchanged	28	n.s.
*PGRN*		33	unchanged	33	n.s.
*PPARγ*		29	unchanged	28	n.s.
Lipases					
*ATGL*		27	unchanged	29	n.s.
*HSL*		30	unchanged	29	n.s.
M1 polarization marker					
*MCP-1*		32	decreased	31	0.013 *
*TNFα*		34	unchanged	33	n.s.
*IL-6*		31	unchanged	29	n.s.
*RANTES*		32	unchanged	32	n.s.
*CD80*		27	unchanged	28	n.s.
M2 polarization marker					
*CD206*		26	increased	27	0.005 *
*Arginase-1*		27	unchanged	25	n.s.
*IL-10*		27	unchanged	28	n.s.

Adipokine serum levels were quantified by ELISA. Gene expression levels were determined by real-time PCR and were normalized to *GAPDH* mRNA levels. *ATGL*, adipose triglyceride lipase; *CD80*, cluster of differentiation 80 (immune-globulin superfamily, expressed on monocytes, antigen-presenting cells, dendritic cells, B-cells); *CD206*, cluster of differentiation 206 (M2 macrophage marker); CTRP-3, C1q/TNF-related protein-3; ELISA, enzyme-linked immunosorbent assay; *HSL*, hormone-sensitive lipase; *IL-6/-10*, interleukin-6/-10; KO, knockout; *MCP-1*, monocyte chemoattractant protein-1; *PGRN*, progranulin. *PPARγ*, peroxisome proliferator-activated receptor gamma; *RANTES*, CC motif chemokine ligand-5; *TNFα*, tumor necrosis factor alpha. * indicates statistical significance. Mean values ± SD are given for quantitative biometric parameters and adipokine levels. Gene expression levels were normalized to *GAPDH* expression and relative changes between control and KO mice were tested for statistical significance.

**Table 2:** Comparative (control vs. CTRP-3 KO mice) transcriptome sequencing by RNA-Seq of primary mature adipocytes (isolated from intra-abdominal adipose tissue) (**A**) and total liver tissue (**B**).

**A**					
**Upregulated Genes**					
**Stable_id**	**Name**	**Description**	**log2FC**	***p* Value**	**fdr**
ENSMUSG00000061911	*Myt1l*	myelin transcription factor 1-like [Source:MGI Symbol;Acc:MGI:1100511]	1.307465982	3.00303 × 10^–11^	4.6544 × 10^–7^
ENSMUSG00000076258	*Gm23935*	predicted gene, 23935 [Source:MGI Symbol;Acc:MGI:5453712]	0.987707951	7.74724 × 10^–10^	6.00373 × 10^–6^
ENSMUSG00000087943	*Gm24245*	predicted gene, 24245 [Source:MGI Symbol;Acc:MGI:5454022]	0.955497729	1.47437 × 10^–8^	7.61711 × 10^–5^
ENSMUSG00000096887	*Gm20594*	predicted gene, 20594 [Source:MGI Symbol;Acc:MGI:5295700]	0.895398238	1.35443 × 10^–5^	0.029989107
ENSMUSG00000086324	*Gm15564*	predicted gene 15564 [Source:MGI Symbol;Acc:MGI:3783013]	0.865783185	3.09707 × 10^–6^	0.009600304
ENSMUSG00000076281	*Gm24270*	predicted gene, 24270 [Source:MGI Symbol;Acc:MGI:5454047]	0.813376051	4.23787 × 10^–6^	0.010947126
ENSMUSG00000088246	*Gm25911*	predicted gene, 25911 [Source:MGI Symbol;Acc:MGI:5455688]	0.77894899	1.97157 × 10^–5^	0.038196634
**Downregulated Genes**					
**Stable_id**	**Name**	**Description**	**log2FC**	***p* Value**	**fdr**
ENSMUSG00000026107	*Nabp1*	nucleic acid binding protein 1 [Source:MGI Symbol;Acc:MGI:1923258]	−0.691646461	2.44321 × 10^–6^	0.009466828
**B**					
**Upregulated Genes**					
**Stable_id**	**Name**	**Description**	**log2FC**	***p* Value**	**fdr**
ENSMUSG00000041293	*Adgrf1*	adhesion G protein-coupled receptor F1 [Source:MGI Symbol;Acc:MGI:1924846]	1.239856554	1.13594 × 10^–9^	7.16403 × 10^–6^
ENSMUSG00000020122	*Egfr*	epidermal growth factor receptor [Source:MGI Symbol;Acc:MGI:95294]	0.829495923	3.30804 × 10^–5^	0.029803838
**Downregulated Genes**					
**Stable_id**	**Name**	**Description**	**log2FC**	***p* Value**	**fdr**
ENSMUSG00000022129	*Dct*	dopachrome tautomerase [Source:MGI Symbol;Acc:MGI:102563]	−0.822078018	5.91212 × 10^–7^	0.001623412
ENSMUSG00000012187	*Mogat1*	monoacylglycerol O-acyltransferase 1 [Source:MGI Symbol;Acc:MGI:1915643]	−0.847754458	2.81302 × 10^–5^	0.027588471
ENSMUSG00000073460	*Pnldc1*	poly(A)-specific ribonuclease (PARN)-like domain containing 1 [Source:MGI Symbol;Acc:MGI:2685159]	−0.852074869	7.12385 × 10^–6^	0.008985543
ENSMUSG00000061780	*Cfd*	complement factor D (adipsin) [Source:MGI Symbol;Acc:MGI:87931]	−0.897079711	4.32255 × 10^–6^	0.006290967
ENSMUSG00000022096	*Hr*	hairless [Source:MGI Symbol;Acc:MGI:96223]	−0.93125267	9.04715 × 10^–7^	0.001623412
ENSMUSG00000089665	*Fcor*	Foxo1 corepressor [Source:MGI Symbol;Acc:MGI:1915484]	−0.952767753	9.43844 × 10^–7^	0.001623412
ENSMUSG00000012123	*Aim1l*	absent in melanoma 1-like [Source:MGI Symbol;Acc:MGI:1334463]	−0.975100738	1.54815 × 10^–7^	0.00058582
ENSMUSG00000002831	*Plin4*	perilipin 4 [Source:MGI Symbol;Acc:MGI:1929709]	−0.999361647	7.5625 × 10^–7^	0.001623412
ENSMUSG00000034634	*Ly6d*	lymphocyte antigen 6 complex, locus D [Source:MGI Symbol;Acc:MGI:96881]	−1.007058408	7.44224 × 10^–7^	0.001623412
ENSMUSG00000046415	*B430212C06Rik*	RIKEN cDNA B430212C06 gene [Source:MGI Symbol;Acc:MGI:2442134]	−1.108254865	7.43126 × 10^–9^	3.51498 × 10^–5^
ENSMUSG00000039092	*Sptlc3*	serine palmitoyltransferase, long chain base subunit 3 [Source:MGI Symbol;Acc:MGI:2444678]	−1.247137857	2.08131 × 10^–10^	1.96892 × 10^–6^

Genes were considered as differentially regulated in case of an absolute value of log_2_ FC (log_2_ fold change) > 0.5 and a corrected *p* value (false discovery rate, fdr; Benjamini–Hochberg correction) < 0.05. For liver tissue samples, only results with log_2_ FC > 0.8 are shown for reasons of better clarity. ENSMUSG, Ensembl *Mus musculus* reference genome; MGI, mouse genome informatics database.

**Table 3:** Primer pairs for gene expression analysis in murine adipocytes and adipose tissue.

Target Gene	Forward Primer 5′–3′	Reverse Primer 5′–3′
*Adiponectin*	AGGGAGAGAAAGGAGATGCAG	CAGACTTGGGCTCCCACCTC
*Arg1*	TTTTAGGGTTACGGCCGGTG	CCTCGAGGCTGTCCTTTTGA
*Ascl1*	GGAACAAGAGCTGCTGGACT	GTTTTTCTGCCTCCCCATTT
*ATGL*	GAGGAATGGCCTACTGAACC	AGGCTGCAATTGATCCTCCT
*CD80*	TCTGTAAGCACAGAAGCTGTTT	GGCTTCACCTAGAGAACCGT
*CD206*	GGCTGATTACGAGCAGTGGA	CATCACTCCAGGTGAACCCC
*GAPDH*	TGTCCGTCGTGGATCTGAC	AGGGAGATGCTCAGTGTTGG
*HSL*	TGAGATGGTAACTGTGAGCC	ACTGAGATTGAGGTGCTGTC
*IL6*	AGTTGCCTTCTTGGGACTGA	TCCACGATTTCCCAGAGAAC
*IL10*	ATTTGAATTCCCTGGGTGAGAAG	CACAGGGGAGAAATCGATGACA
*Leptin*	AGGATCTGAGGGGTGATGTG	AGGTGACCAAGGTGGCATAG
*MCP-1*	AGGTCCCTGTCATGCTTCTG	TCTGGACCCATTCCTTCTTG
*Myt1l*	CGAGCCAGCAACGGTATAGA	CCATTGTGGTGATCTGAGTTCTG
*RANTES*	CCACTTCTTCTCTGGGTTGG	GTGCCCACGTCAAGGAGTAT
*Resistin*	TGCTAAGTCCTCTGCCACGTA	TCAACTGACCGACATCAGGA
*PPARγ*	TTATAGCTGTCATTATTCTCAGTGGAG	CAGCACCTGCCTTAAGTTGA
*Progranulin*	GTGGCTGGCCTGGAGAAGAT	CCTCACAGCACACAGCATGG
*TNFα*	CCAGACCCTCACACTCAGATCAT	ACTCCAGCTGCTCCTCCACTT

## Data Availability

The data presented in this study are available on request from the corresponding author.
